# Detection of concealed structural heart disease by imaging in patients with apparently idiopathic premature ventricular complexes: A review of current literature

**DOI:** 10.1002/clc.23271

**Published:** 2019-09-30

**Authors:** Biagio Sassone, Daniele Muser, Michela Casella, Mario Luzi, Santo Virzì, Cristina Balla, Gaetano Nucifora, for the Task Force on Imaging and Task Force on Ablation of Ventricular Tachycardia of the Italian Association of Arrhythmias and Cardiac Pacing (AIAC)

**Affiliations:** ^1^ Cardiology Division, SS.ma Annunziata Hospital, Department of Emergency Azienda Unità Sanitaria Locale di Ferrara Ferrara Italy; ^2^ Cardiology Division, Delta Hospital, Department of Emergency Azienda Unità Sanitaria Locale di Ferrara Ferrara Italy; ^3^ Cardiothoracic Department, University Hospital Piazzale Santa Maria della Misericordia 15 Udine Italy; ^4^ Heart Rhythm Center Centro Cardiologico Monzino Milan Italy; ^5^ Cardiology Division Ospedale Provinciale AREA VASTA 3 Macerata MC Italy; ^6^ Cardiology Department, S. Anna Hospital University of Ferrara Ferrara Italy; ^7^ Cardiology Department, Wythenshawe Hospital Manchester University NHS Foundation Trust Wythenshawe UK

**Keywords:** cardiac magnetic resonance, premature ventricular complexes, risk stratification

## Abstract

**Background:**

Premature ventricular complexes (PVCs) are the most common form of ventricular arrhythmia in the general population. While in most cases PVCs represent a primitive phenomenon with benign behavior, in a non‐negligible proportion of subjects frequent PVCs may be epiphenomenon of underlying occult heart diseases, requiring special medical attention since they have been resulted linked to increased total and cardiac mortality. Nevertheless, PVCs themselves, when incessantly frequent, may be responsible for left ventricular dysfunction in otherwise normal heart. Aim of this narrative review is to update current knowledge on the general approach to patients with frequent PVCs on the basis of available data, with a special focus on the value of imaging.

**Hypothesis:**

Routine diagnostic work‐up not infrequently miss subtle concealed arrhythmic substrate, leading to erroneously refer to such arrhythmias as to “idiopathic”.

**Methods:**

Literature search of PVCs articles was conducted in PubMed and Scopus electronic database.

**Results:**

Conflicting data arise from literature about the true clinical significance of idiopathic PVCs. There is growing body of data providing evidence that more advanced non‐invasive imaging modalities, such as cardiac magnetic resonance, have an incremental diagnostic and prognostic value. On the other hand, in some cases the prognostic significance of isolated subtle myocardial structural abnormalities in patients with PVCs, still remains area of uncertainty.

**Conclusion:**

In selected subjects with PVCs and high‐risk features for concealed arrhythmic substrate, traditional assessment to rule out the presence of heart disease, including surface ECG and transthoracic echocardiography, should be implemented with more advanced cardiovascular imaging modalities.

## 1 INTRODUCTION

In the vast majority of patients, premature ventricular complexes (PVCs) arise in the absence of underlying structural heart disease (SHD), inherited primary arrhythmia syndromes or metabolic disturbances. In these patients, PVCs are referred as to “idiopathic”, are usually benign in nature and do not require specific treatment. However, in a minority of patients, idiopathic PVCs have been reported as responsible for triggering malignant ventricular arrhythmias or, in case of high arrhythmic burden, the development of left ventricular (LV) dysfunction. Moreover, PVCs may be epiphenomenon of concealed SHD, such as healed myocardial infarction, chronic myocarditis and non‐ischemic cardiomyopathies that may remain undiagnosed after the initial assessment. In this setting, PVCs carry a higher risk of adverse cardiovascular events including sudden cardiac death (SCD).^1^ In the last decade, a widespread use of advanced cardiac imaging modalities such as cardiac magnetic resonance (CMR), have improved our diagnostic accuracy. Aim of this narrative review was to update current knowledge on the general approach to patients presenting with frequent PVCs, with a special focus on the value of imaging, on the basis of available data. Published articles in English language were initially selected by searching the following headings on PubMed and Scopus electronic database: premature ventricular complexes (or synonyms) and idiopathic, risk stratification, cardiomyopathy, cardiac magnetic resonance or ablation. Title and abstract were preliminary screened and potentially relevant reports were then retrieved as complete manuscripts. References lists of selected articles were reviewed for further relevant articles. Pediatric and animal studies were excluded.

## 2 EPIDEMIOLOGY

The prevalence of frequent PVCs in the general population ranges between less than 5% to up to 30% in larger studies with a slightly higher prevalence in males compared to females and a progressive increase with age, being infrequent (<1%) in healthy children <11 year old and very common (>69%) in elderly subjects >75 year old.^1^, ^2^ This variability is largely driven by the different methods used to identify PVCs. In the prospective Atherosclerosis Risk in Communities study, a 2‐minute ECG‐strip documented presence of PVCs in 6% of middle‐aged adults without history of coronary artery disease while Kostis et al. reported a 10% prevalence of more than 100 PVCs/day on a 72‐hour ECG monitoring among subjects without SHD on the basis of non‐invasive and invasive screening, including heart catheterization and coronary angiography.^3^, ^4^

## 3 PROGNOSIS

Data on the prognostic significance of PVCs are conflicting with several studies reporting an increased risk of heart failure and cardiovascular death while others reporting a very low incidence of major adverse cardiac events even on very long‐term follow‐up.^1^, ^2^, ^5^ Several demographic and electrocardiographic factors including male gender, older age, PVC burden, presence of multifocal PVCs and PVC‐QRS duration have been correlated with an increased risk of adverse events, with the main prognostic element being represented by the presence of underlying SHD.

The wide range of criteria used to rule out the presence of heart disease partially justifies the variability of outcomes reported in population‐based studies. Indeed, early studies relied only on medical history, physical examination and standard ECG to define PVCs as “idiopathic”. A recent meta‐analysis summarizing the evidence from 11 population‐based studies on the specific setting of frequent PVCs, arbitrarily defined as ≥1 PVC on standard 12‐lead ECG or ≥30 PVCs on 1‐hour monitoring, found a 3‐fold increased risk of SCD and a 2‐fold increased risk of cardiac death associated with presence of PVCs but these conclusions should be carefully weighted considering the lack of restrictive criteria to identify the presence of SHD in most of the studies included in the analysis.^5^ Recent evidence suggests that even a more accurate screening with surface ECG and transthoracic echocardiography (TTE) may not be sensible enough to detect subtle myocardial structural abnormalities.^6^ Indeed, when the diagnostic work‐up was implemented with CMR imaging, presence of abnormal myocardial findings could be as high as 50%.^7^ Interestingly, when PVCs were identified as truly idiopathic on the basis of accurate screening, they no longer predicted mortality or SCD.^8^ Aquaro et al reported presence of right ventricular (RV) abnormalities detected by CMR in 126 out of 396 (32%) patients with apparently idiopathic RV outflow tract (RVOT) PVCs. RV abnormalities were associated with a 32‐fold increase risk of major arrhythmic events while only 1 event was observed among the remaining 270 patients with normal CMR findings. Of note, PVC burden on 24‐hour Holter monitoring was neither a specific marker of concealed SHD nor a reliable predictor of adverse outcomes.^9^ A potential role for risk stratification of invasive electrophysiological testing has been recently proposed by Yokokawa et al in a large series of patients undergoing catheter ablation (CA) of PVCs, in which the combination of CMR myocardial abnormalities with inducibility of sustained ventricular tachycardia (VT) at programmed ventricular stimulation was associated with a 26‐fold increased risk of adverse outcomes compared to only a 2‐fold increased risk conferred by abnormal CMR findings alone.^10^ Of note, in rare cases even truly idiopathic PVCs may be related to adverse outcomes such as fatal arrhythmias or development of cardiomyopathy.

## 4 MALIGNANT VENTRICULAR ARRHYTHMIAS TRIGGERED BY IDIOPATHIC PVCS

In patients without heart disease, the most typical form of sustained ventricular arrhythmia initiated by PVCs is monomorphic VT originating from ventricular outflow tract. This form of sustained VT have been reported as being benign in most cases. However, PVCs triggering threatening ventricular arrhythmias, such as ventricular fibrillation (VF) or polymorphic ventricular tachycardia (PVT), have also been reported in patients with normal heart.^11^, ^12^ In these setting the onset of VF seems to be related to a vulnerability of the Purkinje network with triggering monomorphic PVCs arising from the RVOT, the papillary muscles or the moderator band in up to 75% of the cases. In these cases CA of the triggering PVC has been proved to be highly effective in preventing VF recurrence on long‐term follow‐up.^13^ Several studies have associated PVC‐triggered VF to an history of previous unexplained syncope in almost 80% of the cases compared to 18%‐39% of patients with RVOT PVCs and sustained VT of the same morphology, making healthy subjects with PVCs and history of syncope at particular high risk and deserving careful medical attention.^12^ In some series PVCs triggering VF have been linked to a shorter coupling interval but these results have not been subsequently confirmed making substantially impossible to identify the potential malign nature of PVCs on the basis of their coupling interval.^14^

## 5 PVC‐INDUCED CARDIOMYOPATHY

Longstanding frequent PVCs may be the cause of LV impairment in up to 7% of cases of otherwise unexplained dilated cardiomyopathy.^15^ The development of LV adverse remodeling and dysfunction requires several years before becoming clinically manifest, thus patients with persistent frequent PVCs should be annually monitored for LV function by TTE, even if completely asymptomatic. A daily PVC burden between 16% and 24% of all heartbeats have been associated to progressive LV dysfunction.^16^ However, subtle myocardial mechanical dysfunction detected by speckle tracking echocardiography has been reported also in patients with a PVC burden as low as 4%.^17^ Left bundle‐branch block (LBBB) PVC‐QRS morphology and longer PVC‐QRS duration (>150 ms) as well as an epicardial origin of PVCs require a lower arrhythmic burden to develop LV dysfunction, thus corroborating the hypothesis of mechanical dyssynchrony as the main mechanism of PVC‐induced cardiomyopathy.^18^ Accordingly, narrower PVCs originating from endocardial sites close to the Purkinje network such as the basal septum or left bundle branch fascicles, are rarely responsible for LV dysfunction owing to the less dyssynchronous effect upon ventricular contraction. In patients presenting with frequent PVCs and LV dysfunction, differentiating between reversible PVC‐induced cardiomyopathy and dilated cardiomyopathy manifesting with frequent PVCs is not always easy and typically a definitive diagnosis is possible only on retrospective basis by demonstration of reversible LV dysfunction after effective suppression of PVCs by anti‐arrhythmic drugs (AADs) or CA.^19^ In this regard, several factors have been linked with reversible cardiomyopathy after successful treatment of PVCs including a pre‐treatment PVC burden of at least 10% and absence of myocardial scar detectable by late gadolinium enhancement (LGE) CMR.^20^ A complete recovery of LV ejection fraction is typically observed within 4 months in patients with PVC‐induced cardiomyopathy in whom a complete suppression or at least an 80% reduction of PVC burden is obtained.^21^

## 6 DIAGNOSTIC WORKUP OF PATIENTS WITH FREQUENT PVCS

### 6.1 Initial evaluation

Initial patient evaluation should always include detailed clinical history taking concerning personal and family history of inherited arrhythmic syndromes, cardiomyopathies or SCD. While the regular consumption of some drugs (ie, ß‐agonists, thyroid hormones, amphetamines) has a definite linkage with PVCs occurrence, this has not been established definitively with the habitual consumption of adrenergic substances such as caffeine, alcohol and tobacco.^22^ A panel of laboratory tests may be helpful to rule out metabolic disorders potentially triggering cardiac arrhythmias such as hyperthyroidism. Because careful evaluation of surface 12‐lead ECG may provide information about arrhythmia substrates or triggers, diagnostic workup should routinely include it; then, if any suspicion of SHD arises, TTE and exercise stress test should be also considered as complementary investigations. Idiopathic PVCs are typically related to triggered activity due to cyclic adenosine monophosphate‐mediated afterdepolarizations, are frequently monomorphic and originate from the right and left ventricular outflow tracts in up to 70% of the cases, thus presenting with a LBBB QRS morphology and an inferiorly directed frontal axis.^23^ Conversely, PVCs related to SHD typically have a re‐entry mechanism, are more frequently multifocal and often present a RBBB QRS morphology with a superiorly directed axis.^24^, ^25^ At least in some cases ECG depolarization and repolarization abnormalities such as inverted T waves are present and consistent with presence of wall motion abnormalities on TTE.^25^, ^26^ Exercise stress testing is pivotal in risk stratification of patients with frequent PVCs. In a French study including more than 6000 asymptomatic middle‐age men without history of SHD, frequent PVCs during bicycle exercise testing, occurred in 2.3% of all subjects and resulted independently associated with an increased risk of cardiovascular death by a factor of 2.5 during long‐term follow‐up. Interestingly, only 5.8% of patients with exercise‐induced PVCs had ECG findings suggestive for myocardial ischemia during stress.^27^ Eckart et al also reported an higher prevalence of fixed‐perfusion defects consistent with past myocardial infarction or scarring among patients with exercise‐induced frequent PVCs referred for ^99m^Tc‐sestamibi myocardial stress perfusion imaging compared to patients without arrhythmias. Interestingly, only exercise‐induced multifocal PVCs or PVCs with RBBB morphology were associated with higher risk for death in this series.^28^ In a recent CMR study, 68% of 162 patients with exercise‐induced PVCs without SHD on routine diagnostic work up had evidence of areas of LGE with a sub‐epicardial or mid‐myocardial distribution consistent with previous myocarditis, compared to only 9% of controls. Moreover, in 37% of such cases myocardial oedema consistent with acute inflammatory process was also detected.^29^ In a large cohort of 29 244 asymptomatic patients without history SHD who underwent treadmill exercise testing, frequent PCVs occurring during the recovery‐phase (≥7 PVCs/min) were detected in 3.7% of cases and were associated with a 2.4‐fold increase risk of death at 5‐years follow‐up. Of note, PVCs during recovery‐phase were a stronger predictor of increased mortality than PVCs occurring only during exercise and resulted associated with higher rate of myocardial ischemia during exercise‐stress echocardiography.^30^

### 6.2 Cardiac magnetic resonance

**Figure 1:**
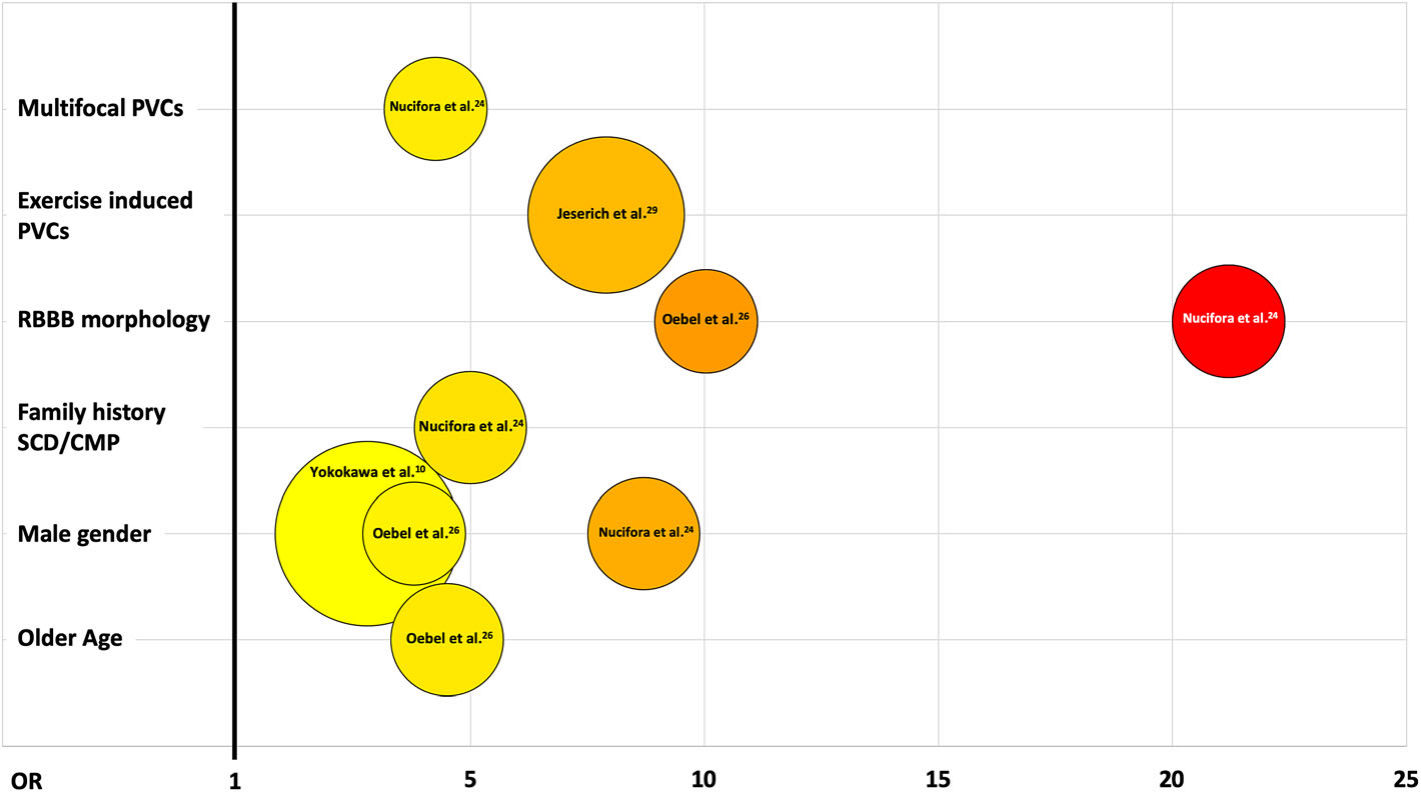
Predictors of cardiac magnetic resonance abnormalities in patients with apparently idiopathic premature ventricular complexes (PVCs). The odds‐ratio of each factor is indicated on the horizontal axis and color coded within the circles; the area of each circle is proportional to the numerosity of the sample investigated. CMP: cardiomyopathy; RBBB: right bundle branch block; SCD: sudden cardiac death [Correction added on 14th October 2019, after first online publication: Figure 1 replaced with updated figure, which includes updated references.]

**Figure 2:**
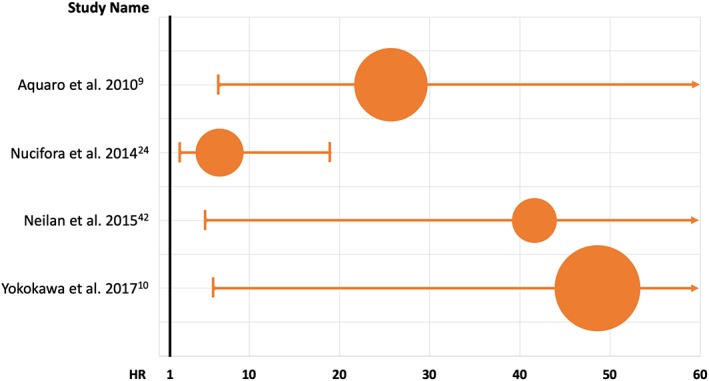
Forest plot showing the results of principal studies investigating the risk of major adverse cardiovascular events associated with the presence of myocardial structural abnormalities detected by cardiac magnetic resonance in patients with apparently idiopathic ventricular arrhythmias [Correction added on 14th October 2019, after first online publication: Figure 2 replaced with updated figure, which includes updated references.]

CMR currently represents the most accurate non‐invasive imaging technique for identification and characterization of myocardial arrhythmogenic substrate. CMR imaging is able to accurately and reproducibly quantify biventricular volumes and function, and to characterize myocardial tissue by detecting presence of myocardial fatty replacement, myocardial oedema and necrosis/fibrosis, especially regarding the RV structure whose subtle alterations may otherwise remain unrecognized at initial diagnostic work up or with other imaging modalities.^7^, ^31^ Focal myocardial abnormalities representing the result of local inflammatory processes (eg, healed myocarditis) or the early manifestation of a cardiomyopathy have been described in up to half of the patients with frequent PVCs and unremarkable ECG and echocardiographic findings.^7^, ^24^, ^31^ Altogether, studies that have investigated CMR findings in patients with frequent PVCs have found a pool of baseline clinical variables and arrhythmia features to be correlated with presence of myocardial structural abnormalities (Figure 1). In most of these cases CMR reveals one or more foci of non‐ischemic LGE more frequently located within the septum or the LV lateral wall.^25^ The identification of subtle myocardial abnormalities has a significant clinical impact, as they have been correlated with an increased risk of malignant arrhythmic events during the long‐term follow‐up.^25^ Multiple studies have investigated the prognostic role of CMR imaging in patients presenting with ventricular arrhythmias (VAs) and repeatedly reported presence of LGE to be an independent predictor of adverse outcomes regardless the underlying LV ejection fraction.^32^ Even if the vast majority of such studies have included an heterogeneous population of patients with different forms of VAs (including sustained VT/VF), similar findings, showing a relationship between CMR features and prognosis, have been reported in few studies specifically addressing patients with isolated PVCs (Figure 2).

### 6.3 Cardiac computed tomography

ECG‐triggered or gated multislice CT (MSCT) represents a useful imaging modality to non‐invasively assess the origin and course of the coronary arteries and the presence of coronary artery disease, due to its high‐spatial resolution and the capability of multiplanar reconstructions. Also, in cardiomyopathy subjects without obstructed coronary arteries and with PVCs, MSCT is able to identify areas of myocardial fibrosis potentially related to poor outcome.^33^ Advances in CT technology observed in the last decade allow also to minimize radiation exposure. However, frequent PVCs and suboptimal heart rate control significantly reduce the ability to acquire diagnostic datasets and increase the risk of higher radiation dose.

### 6.4 Nuclear medicine

Nuclear imaging, especially positron emission tomography (PET), is acquiring a growing role in the management of patients presenting with VAs. Using different tracers, PET is able to assess myocardial perfusion, presence of inflammation, myocardial viability and sympathetic innervation which all may have a role in the genesis and maintenance of VAs.^34^ So far only two studies have applied ^18^F‐fluorodeoxyglucose (^18^F‐FDG) PET to investigate the presence of subclinical myocardial inflammation in patients with VAs.^34^ In one of them, abnormal ^18^F‐FDG uptake was found in up to 50% of patients presenting with VAs and non‐ischemic cardiomyopathy of unknown etiology.^35^ More recently, a single center prospective study (Myocarditis and Ventricular Arrhythmia registry—MAVERIC) including patients with frequent PVCs and no SHD on the basis of ECG and TTE, reported abnormal myocardial ^18^F‐FDG uptake in 51% of patients. Patients treated with immunosuppression due to positive PET, demonstrated a good clinical response with a significant decrease in PVC burden in up to 80% of the cases.^34^ Even if the above data seem promising, the application of ^18^F‐FDG PET in daily clinical practice as well as the decision to treat patients with immunosuppression should be carefully weighted and needs further validation by randomized controlled studies.

**Figure 3:**
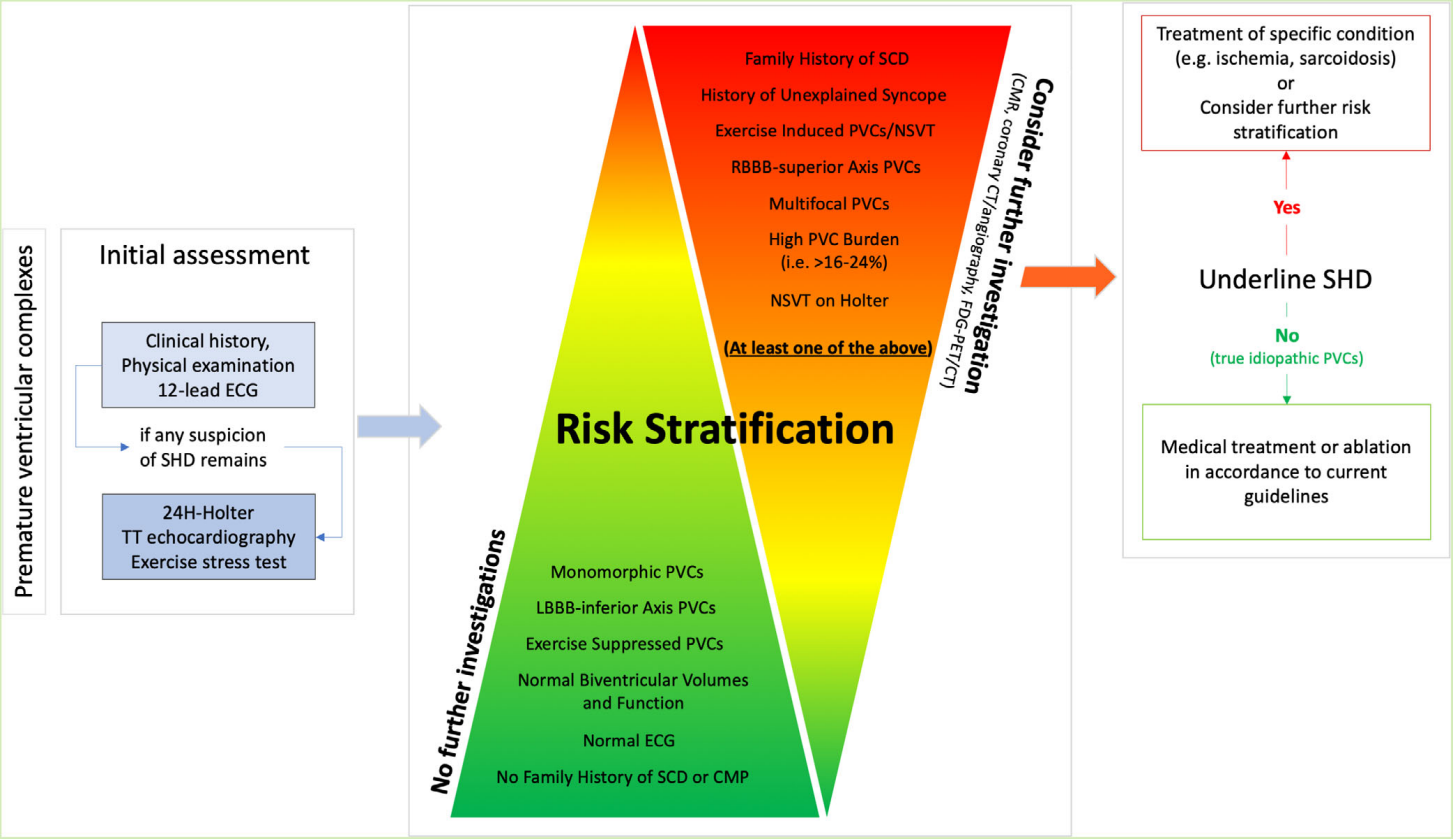
Proposed flow‐chart for the diagnostic work‐up and management of patients presenting with apparently idiopathic premature ventricular complexes (PVCs). Careful evaluation of personal history, baseline electrocardiogram (ECG) and PVC features should be always the first step in the diagnostic work‐up of patients with frequent PVCs (left panel); complementary diagnostics may be needed to rule out suspected causes of PVCs (eg, valvular or ischemic heart disease). When features suggestive of truly idiopathic PVCs are present (central panel, green triangle), no further investigations are mandatory. Conversely, when one or more high‐risk features (central panel, red triangle) are present, further investigations with advanced imaging techniques should be considered in order to detect possible underlying structural abnormalities deserving proper medical attention and specific treatment (right panel) CMP: cardiomyopathy; CT: computed tomography; FDG‐PET: fluorodeoxyglucose positron emission tomography; LBBB: left bundle branch block; LV: left ventricle; NSVT: non‐sustained ventricular tachycardia; RBBB: right bundle branch block; SCD: sudden cardiac death; SHD: structural heart disease; TT: transthoracic

When an accurate initial evaluation process detects high‐risk features, thus raising suspicion of concealed SHD, the use of advanced cardiovascular imaging techniques (eg, CMR, MSCT, PET) should be considered. The flow‐chart in Figure 3 offers an easy‐to‐consult graphic model to orientate the decision‐making process throughout a comprehensive diagnostic work‐up and management of patients presenting with frequent PVCs.

### 6.5 Genetic testing in idiopathic PVCs

Current guidelines recommend genetic testing in patients with VAs and/or aborted SCD or in family members of SCD victims when an inheritable arrhythmogenic disease is suspected.^36^ No specific recommendations are currently known for genetic testing in patients with idiopathic PVC unless clinical features and family history raise the suspicion of an inherited arrhythmic disorder. In the last few decades, genetic testing for channelopathies and cardiomyopathies had advanced from initial scientific discoveries to clinical application in daily clinical practice. Therefore, genetic information may support a suspected clinical diagnosis of the proband, offering a cascade screening of family members when a disease‐causing mutation is identified.^37^ It is important to highlight that genotyping have a direct diagnostic, prognostic and therapeutic implication only in some cases such as long QT syndrome while in other disorders such as Brugada syndrome, it does not provide additional clinical information for the proband. In these setting, genotyping is mostly useful for family screening of a mutation‐positive proband to identify gene positive relatives in a preclinical phase allowing the introduction of lifestyle changes or therapies. On the other hand, genotype‐negative family members may be dismissed from periodic follow up.^37^

### 6.6 Therapy: when and how to treat idiopathic PVCs

In the majority of cases, idiopathic PVCs are a benign clinical condition with a good prognosis and a specific treatment is usually unnecessary. The 2014 EHRA/HRS/APHRS Expert Consensus on VAs suggests, as first step, reassurance about the benign nature of this arrhythmia allaying patients' anxiety.^38^ In case of highly symptomatic patients, significant PVCs burden (with or without symptoms) or suspicion of PVC‐induced cardiomyopathy, a therapeutic intervention is recommended. AADs are commonly used as a first‐line option even though no large‐scale randomized trials on AAD therapy for idiopathic PVCs have been performed.^39^ Usually beta‐blockers or non‐dihydropyridine calcium antagonists are considered as first‐line therapy but their efficacy is quite limited, being similar to placebo, and the occurrence of side effects are not infrequent, thus reducing patient's compliance. While Class I AADs are highly effective to suppress PVCs and improve symptoms, the risk‐benefit ratio has not yet been carefully evaluated in patients without underlying cardiomyopathy. Multiple studies indicate high efficacy of CA with PVC elimination in 74%‐100% of patients associated with low complication rate (about 1%). The only prospective randomized study comparing CA and AADs confirmed the superiority of the ablative approach in preventing arrhythmia recurrences, at least in patients with PVCs originating from RVOT.^39^ Indeed, CA success is strongly related to the PVCs site of origin with RVOT‐PVCs being the most easily approachable. Idiopathic PVC origins different from RVOT, such as LVOT, aortic cusp or epicardial origins or multiple morphologies, have been demonstrated to be associated with a lower success rate and with a trend toward the highest complication rate.^40^ The recent 2019 HRS/EHRA/APHRS/LAHRS Expert Consensus on CA of VAs, recommends CA of idiopathic PVCs in patients with symptomatic PVCs originating from RVOT as first line therapeutic approach in preference to AAD therapy.^41^ In patients with symptomatic PVCs originating from other sites than RVOT (including LVOT, epicardial OT or LV summit) or in those presenting with polymorphic PVCs, CA can be considered only after failed/not tolerated medical therapy or when patients refuse taking drugs.^41^

Owing to the benign nature of truly idiopathic PVCs, implantable cardiac defibrillators are not recommended. However, it should be considered in patients with persistent LV dysfunction after a reasonable waiting period of 4‐6 months following efficacious AAD or ablative treatment, according to the current guideline recommendations for primary prevention of SCD in heart failure.

### 6.7 Economic and organizational issues

Whether all patients with frequent PVCs and apparently normal heart by routine diagnostic tests deserve further investigation with more advanced imaging modalities (eg, CMR imaging) is still unclear. This issue needs to take into account several aspects, including lack of widespread availability and direct and indirect costs of these technologies, reimbursement and sustainability for the healthcare system, needs for appropriate training and education. Identification of those subjects who may benefit most from further investigations appears therefore relevant. Previous studies have identified older age, male gender, family history of SCD/cardiomyopathy and few PVC features (Figure 1) as significantly and independently correlated to the presence of myocardial structural abnormalities by CMR imaging; these baseline characteristics could be proposed as “red flags” to identify those subjects with apparently idiopathic PVCs that may conversely have concealed cardiomyopathic substrates and therefore get the higher benefit from a more comprehensive diagnostic work‐up inclusive of CMR imaging. Organization of a network for referral of patients to high‐volume centers with the adequate equipment and expertise for advanced imaging should be also encouraged.

## 7 CONCLUSIONS

Even if benign in nature in the vast majority of cases, PVCs can sometimes be associated with an increased risk of heart failure and cardiac mortality including SCD. The main challenge in correctly labelling PVCs as benign is the exclusion of subtle myocardial structural abnormalities that can be missed by routine investigations. Thus, in selected subjects with high‐risk features, traditional assessment should be implemented with more advanced imaging modalities such as CMR. On the other hand, the use of more sensitive technologies might rise additional interpretative doubts especially when subtle or isolated myocardial structural abnormalities (eg, fibrosis) are documented in patients with PVCs but without any symptoms or evidence of more complex VAs. As a matter of fact, at least in some cases, subtle myocardial scarring may be an incidental finding (eg, healed myocarditis) without any meaningful relationship with PVC etiology (ie, RVOT PVCs). In such cases, the interaction between PVCs and the potential arrhythmic substrate remains uncertain with a substantial knowledge gap regarding proper risk stratification (eg, invasive electrophysiological study) and therapy.

## CONFLICT OF INTEREST

The authors have declared no conflicting interests. This research did not receive any specific grant from funding agencies in the public, commercial, or not‐for‐profit sectors.

## References

[1] Dukes JW , Dewland TA , Vittinghoff E , et al. Ventricular ectopy as a predictor of heart failure and death. J Am Coll Cardiol. 2015;66:101‐109. 26160626 10.1016/j.jacc.2015.04.062 PMC4499114

[2] Southall DP , Johnston F , Shinebourne EA , Johnston PG . 24‐hour electrocardiographic study of heart rate and rhythm patterns in population of healthy children. Br Heart J. 1981;45:281‐291. 7470341 10.1136/hrt.45.3.281 PMC482524

[3] Simpson RJ , Cascio WE , Schreiner PJ , et al. Prevalence of premature ventricular contractions in a population of African American and white men and women: the atherosclerosis risk in communities (ARIC) study. Am Heart J. 2002;143:535‐540. 11868062 10.1067/mhj.2002.120298

[4] Kostis JB , McCrone K , Moreyra AE , et al. Premature ventricular complexes in the absence of identifiable heart disease. Circulation. 1981;63:1351‐1356. 7226480 10.1161/01.cir.63.6.1351

[5] Ataklte F , Erqou S , Laukkanen J , Kaptoge S . Meta‐analysis of ventricular premature complexes and their relation to cardiac mortality in general populations. Am J Cardiol. 2013;112:1263‐1270. 23927786 10.1016/j.amjcard.2013.05.065

[6] Lee V , Hemingway H , Harb R , Crake T , Lambiase P . The prognostic significance of premature ventricular complexes in adults without clinically apparent heart disease: a meta‐analysis and systematic review. Heart. 2012;98:1290‐1298. 22781425 10.1136/heartjnl-2012-302005

[7] Jeserich M , Friedrich MG , Olschewski M , et al. Evidence for non‐ischemic scarring in patients with ventricular ectopy. Int J Cardiol. 2011;147:482‐484. 21295361 10.1016/j.ijcard.2011.01.055

[8] Niwano S , Wakisaka Y , Niwano H , et al. Prognostic significance of frequent premature ventricular contractions originating from the ventricular outflow tract in patients with normal left ventricular function. Heart. 2009;95:1230‐1237. 19429571 10.1136/hrt.2008.159558

[9] Aquaro GD , Pingitore A , Strata E , di Bella G , Molinaro S , Lombardi M . Cardiac magnetic resonance predicts outcome in patients with premature ventricular complexes of left bundle branch block morphology. J Am Coll Cardiol. 2010;56:1235‐1243. 20883930 10.1016/j.jacc.2010.03.087

[10] Yokokawa M , Siontis KC , Kim HM , et al. Value of cardiac magnetic resonance imaging and programmed ventricular stimulation in patients with frequent premature ventricular complexes undergoing radiofrequency ablation. Heart Rhythm. 2017;14:1695‐1701. 28688990 10.1016/j.hrthm.2017.06.040

[11] Viskin S , Rosso R , Rogowski O , et al. The “short‐coupled” variant of right ventricular outflow ventricular tachycardia: a not‐so‐benign form of benign ventricular tachycardia? J Cardiovasc Electrophysiol. 2005;16:912‐916. 16101636 10.1111/j.1540-8167.2005.50040.x

[12] Noda T , Shimizu W , Taguchi A , et al. Malignant entity of idiopathic ventricular fibrillation and polymorphic ventricular tachycardia initiated by premature extrasystoles originating from the right ventricular outflow tract. J Am Coll Cardiol. 2005;46:1288‐1294. 16198845 10.1016/j.jacc.2005.05.077

[13] Takatsuki S , Mitamura H , Ogawa S . Catheter ablation of a monofocal premature ventricular complex triggering idiopathic ventricular fibrillation. Heart Br Card Soc. 2001;86:E3‐E33. 10.1136/heart.86.1.e3 PMC1729809 11410580

[14] Igarashi M , Tada H , Kurosaki K , et al. Electrocardiographic determinants of the polymorphic QRS morphology in idiopathic right ventricular outflow tract tachycardia. J Cardiovasc Electrophysiol. 2012;23:521‐526. 22136173 10.1111/j.1540-8167.2011.02232.x

[15] Yokokawa M , Kim HM , Good E , et al. Relation of symptoms and symptom duration to premature ventricular complex‐induced cardiomyopathy. Heart Rhythm. 2012;9:92‐95. 21855522 10.1016/j.hrthm.2011.08.015

[16] Saurav A , Smer A , Abuzaid A , Bansal O , Abuissa H . Premature ventricular contraction‐induced cardiomyopathy: PVC‐induced cardiomyopathy. Clin Cardiol. 2015;38:251‐258. 25678299 10.1002/clc.22371 PMC6711062

[17] Lie ØH , Saberniak J , Dejgaard LA , et al. Lower than expected burden of premature ventricular contractions impairs myocardial function. ESC Heart Fail. 2017;4:585‐594. 29154430 10.1002/ehf2.12180 PMC5695171

[18] Carballeira Pol L , Deyell MW , Frankel DS , et al. Ventricular premature depolarization QRS duration as a new marker of risk for the development of ventricular premature depolarization‐induced cardiomyopathy. Heart Rhythm. 2014;11:299‐306. 24184787 10.1016/j.hrthm.2013.10.055

[19] Yarlagadda RK , Iwai S , Stein KM , et al. Reversal of cardiomyopathy in patients with repetitive monomorphic ventricular ectopy originating from the right ventricular outflow tract. Circulation. 2005;112:1092‐1097. 16103234 10.1161/CIRCULATIONAHA.105.546432

[20] Baman TS , Lange DC , Ilg KJ , et al. Relationship between burden of premature ventricular complexes and left ventricular function. Heart Rhythm. 2010;7:865‐869. 20348027 10.1016/j.hrthm.2010.03.036

[21] Yokokawa M , Kim HM , Good E , et al. Impact of QRS duration of frequent premature ventricular complexes on the development of cardiomyopathy. Heart Rhythm. 2012;9:1460‐1464. 22542704 10.1016/j.hrthm.2012.04.036

[22] DeBacker G , Jacobs D , Prineas R , et al. Ventricular premature contractions: a randomized non‐drug intervention trial in normal men. Circulation. 1979;59:762‐769. 421317 10.1161/01.cir.59.4.762

[23] Lerman BB . Mechanism, diagnosis, and treatment of outflow tract tachycardia. Nat Rev Cardiol. 2015;12:597‐608. 26283265 10.1038/nrcardio.2015.121

[24] Nucifora G , Muser D , Masci PG , et al. Prevalence and prognostic value of concealed structural abnormalities in patients with apparently idiopathic ventricular arrhythmias of left versus right ventricular origin a magnetic resonance imaging study. Circ Arrhythm Electrophysiol. 2014;7:456‐462. 24771543 10.1161/CIRCEP.113.001172

[25] Zorzi A , Marra MP , Rigato I , et al. Nonischemic left ventricular scar as a substrate of life‐threatening ventricular arrhythmias and sudden cardiac death in competitive athletes. Circ Arrhythm Electrophysiol. 2016;9:e004229. 27390211 10.1161/CIRCEP.116.004229 PMC4956679

[26] Oebel S , Dinov B , Arya A , et al. ECG morphology of premature ventricular contractions predicts the presence of myocardial fibrotic substrate on cardiac magnetic resonance imaging in patients undergoing ablation. J Cardiovasc Electrophysiol. 2017;28:1316‐1323. 28791747 10.1111/jce.13309

[27] Jouven X , Zureik M , Desnos M , Courbon D , Ducimetière P . Long‐term outcome in asymptomatic men with exercise‐induced premature ventricular depolarizations. N Engl J Med. 2000;343:826‐833. 10995861 10.1056/NEJM200009213431201

[28] Eckart RE , Field ME , Hruczkowski TW , et al.: Association of electrocardiographic morphology of exercise‐induced ventricular arrhythmia with mortality. Ann Intern Med 2008; 149:451–460, W82. 18838725 10.7326/0003-4819-149-7-200810070-00005

[29] Jeserich M , Merkely B , Olschewski M , Kimmel S , Pavlik G , Bode C : Patients with exercise‐associated ventricular ectopy present evidence of myocarditis. J Cardiovasc Magn Reson [Internet] 2015 [cited 2018 Feb 2]; 17 Available from: http://jcmr-online.com/content/17/1/100 10.1186/s12968-015-0204-3 PMC4655086 26590904

[30] Frolkis JP , Pothier CE , Blackstone EH , Lauer MS . Frequent ventricular ectopy after exercise as a predictor of death. N Engl J Med. 2003;348:781‐790. 12606732 10.1056/NEJMoa022353

[31] Mavrogeni S , Anastasakis A , Sfendouraki E , et al. Ventricular tachycardia in patients with family history of sudden cardiac death, normal coronaries and normal ventricular function. Can cardiac magnetic resonance add to diagnosis? Int J Cardiol. 2013;168:1532‐1533. 23276393 10.1016/j.ijcard.2012.12.023

[32] Dawson DK , Hawlisch K , Prescott G , et al. Prognostic role of CMR in patients presenting with ventricular arrhythmias. JACC Cardiovasc Imaging. 2013;6:335‐344. 23433931 10.1016/j.jcmg.2012.09.012

[33] Ozawa K , Funabashi N , Takaoka H , Ueda M , Kobayashi Y . Risk stratification using a combination of left ventricular fibrosis and number of morphological types of ventricular premature beats in cardiomyopathy subjects without obstructed coronary arteries. Int J Cardiol. 2014;176:236‐239. 25037689 10.1016/j.ijcard.2014.06.070

[34] Muser D , Castro SA , Alavi A , Santangeli P . Potential role of PET in assessing ventricular arrhythmias. PET Clin. 2019;14:281‐291. 30826025 10.1016/j.cpet.2018.12.009

[35] Tung R , Bauer B , Schelbert H , et al. Incidence of abnormal positron emission tomography in patients with unexplained cardiomyopathy and ventricular arrhythmias: the potential role of occult inflammation in arrhythmogenesis. Heart Rhythm. 2015;12:2488‐2498. 26272522 10.1016/j.hrthm.2015.08.014 PMC4656080

[36] Priori SG , Blomström‐Lundqvist C , Mazzanti A , et al. ESC guidelines for the management of patients with ventricular arrhythmias and the prevention of sudden cardiac death: the task force for the Management of Patients with ventricular arrhythmias and the prevention of sudden cardiac death of the European Society of Cardiology (ESC). Endorsed by: Association for European Paediatric and Congenital Cardiology (AEPC). Eur Heart J 2015. 2015;36:2793‐2867. 10.1093/eurheartj/ehv316 26320108

[37] Priori SG , Wilde AA , Horie M , et al. HRS/EHRA/APHRS expert consensus statement on the diagnosis and management of patients with inherited primary arrhythmia syndromes: document endorsed by HRS, EHRA, and APHRS in may 2013 and by ACCF, AHA, PACES, and AEPC in June 2013. Heart Rhythm. 2013;10:1932‐1963. 24011539 10.1016/j.hrthm.2013.05.014

[38] Pedersen CT , Kay GN , Kalman J , et al. EHRA/HRS/APHRS expert consensus on ventricular arrhythmias. Heart Rhythm. 2014;11:e166‐e196. 25179489 10.1016/j.hrthm.2014.07.024

[39] Ling Z , Liu Z , Su L , et al. Radiofrequency ablation versus antiarrhythmic medication for treatment of ventricular premature beats from the right ventricular outflow tract: prospective randomized study. Circ Arrhythm Electrophysiol. 2014;7:237‐243. 24523413 10.1161/CIRCEP.113.000805

[40] Latchamsetty R , Yokokawa M , Morady F , et al. Multicenter outcomes for catheter ablation of idiopathic premature ventricular complexes. JACC Clin Electrophysiol. 2015;1:116‐123. 29759353 10.1016/j.jacep.2015.04.005

[41] Cronin EM, Bogun FM, Maury P, et al. 2019 HRS/EHRA/APHRS/LAHRS expert consensus statement on catheter ablation of ventricular arrhythmias. Heart Rhythm 2019. Available from: https://www.heartrhythmjournal.com/article/S1547-5271(19)30210-3/abstract. 10.1016/j.hrthm.2019.03.002 PMC8453449 31085023

[42] Neilan TG , Farhad H , Mayrhofer T , et al. Late gadolinium enhancement among survivors of sudden cardiac arrest. JACC Cardiovasc Imaging. 2015;8:414‐423. [Correction added on 14th October 2019, after first online publication: Reference 42 added.] 25797123 10.1016/j.jcmg.2014.11.017 PMC4785883

